# Study on the accessibility and affordability of 50 drugs in Wuhan based on the WHO/HAI standardization method

**DOI:** 10.3389/fpubh.2023.1108007

**Published:** 2023-01-27

**Authors:** Zuojun Dong, Shaoya Zhang, Shuaijun Wu, Xintong Xie, Guojun Sun, Xuanyao Yu

**Affiliations:** Institute of Pharmaceutical Preparations, Zhejiang University of Technology, Hangzhou, China

**Keywords:** drugs, public hospitals, retail drugstore, availability, the price, affordable

## Abstract

**Objective:**

To understand the availability and affordability of essential drugs in Wuhan since the implementation of the national essential medicine system, and to provide a basis for the subsequent formulation and improvement of related policies.

**Methods:**

Using the standard survey method jointly developed by the WHO and Health Action International (HAI), a sample of 50 essential drugs was selected to investigate and evaluate their availability and affordability in public medical and health institutions and social retail pharmacies in Wuhan, using six diseases with high clinical morbidity as the targets.

**Results:**

The availability of the original drug and the lowest-priced generic drug in public hospitals is 26.4 and 42.47% respectively, and that in retail pharmacies is 26.8 and 54.4% respectively. The median price ratio of the original drug and the lowest-priced generic drug is 28.71 and 2.23 respectively in public hospitals, and 29.24 and 3.59 respectively in retail pharmacies; In addition to individual drugs, such as omeprazole, others are affordable. The availability of essential drugs in public hospitals in Wuhan is lower than that in social retail pharmacies, and the availability of the lowest-priced generic drugs is much higher than that of original drugs.

**Conclusion:**

The availability of essential drugs in public hospitals in Wuhan is lower than that in social retail pharmacies, and the availability of the lowest-priced generic drugs is much higher than that of original drugs. The price of the original drug is much higher than the international reference price; The price of medicines in public hospitals is lower than that in retail pharmacies;the overall condition of affordability is good, but there is a big gap between the affordability levels of original drugs and generic drugs, and the affordability of original drugs is relatively poor. It is recommended to adjust the relevant policies according to the actual situation of Wuhan city itself, moderately ensure the supply of original drugs, improve the price transparency of retail pharmacies, and ensure that the basic drug needs of the public are met.

## 1. Introduction

The scarcity of resources determines that prioritizing basic needs and improving the efficiency of resource use are necessary at all times, and the concept of essential drugs was born on such a premise. Ensuring the accessibility of essential medicines can help to meet the most basic needs of the public and promote the rational use of medicines to improve the efficiency of the use of health resources. The concept of Essential Medicines was first introduced by WHO in 1975, to provide medicines that meet clinical needs, are in appropriate dosage forms, reasonably priced, securely available, and equitably accessible. The first edition of the WHO Model List of Essential Medicines, also known as the WHO Essential Medicines List, was published in 1977. This list includes the Model List of Essential Medicines and the Model List of Essential Medicines for Children. Access to essential drugs is part of the realization of the right to the highest attainable standard of health, and the basic right to health cannot be realized without equal access to essential medicines for priority diseases. The essential medicines system is one of the more successful global health policies (1).

In 1979, our government participated in the WHO Action Plan for Essential Medicines and set up the National Essential Medicines Selection Group under the organization of the former Ministry of Health and the former State General Administration of Pharmaceuticals to start the formulation of national essential medicines. In 1982, the first National Essential Medicines Catalog was published, which contained 278 medicines and was selected mainly on the principle of clinical necessity to ensure the most basic needs of the people. In 1992, the National Essential Medicines Leading Group was set up to organize and lead the selection and implementation of national essential medicines to coordinate with the reform of the publicly-funded medical care and medical insurance system. In 2009, the construction of the national essential drug system was officially launched, with provinces implementing the essential drug system in 30% of government-run urban community health service institutions and counties (grassroots medical and health institutions), implementing centralized online public tender procurement and unified distribution at the provincial level, and equipping all essential drugs and achieving zero-rate sales (2). The basic drug system is implemented at the provincial level. With reference to international experience, the dosage form and quantity of China's essential drug varieties are reasonably determined, and on the basis of maintaining a relatively stable quantity, the National Essential Drug Catalog is dynamically adjusted and managed, in principle, every 3 years (3). In principle, it will be adjusted every 3 years. In order to further consolidate and improve the essential drug system, the former Ministry of Health and the National Health and Health Commission issued an edition of the National Essential Drug List, National Essential Drug Clinical Application Guidelines, and National Essential Drug Formulary after selection in 2009, 2012, and 2018. On November 15, 2012, the Drug Secretary of the National Health and Health Commission issued an article on the National Essential Drug List Management Measures (Revised Draft) (The draft of the National Essential Medicines Catalog (Revised Draft) was issued by the State Health and Welfare Commission for public consultation, clarifying that the selection of essential medicines is based on the functional positioning of “highlighting the basic, prevention and treatment necessity, guaranteeing supply, prioritizing use, ensuring quality and reducing burden”, adhering to the principles of equal emphasis on Chinese and Western medicines and clinical preference, and reasonably determined with reference to international experience (4). The National Essential Drug List is a basic drug list that meets basic medical and health needs, is reasonably priced, and is fairly accessible to the public. It is a basic catalog to guarantee the basic medical needs of the nation, with more emphasis on accessibility. The reimbursement ratio of drugs in the basic drug catalog is significantly higher than that of non-basic drugs, which can effectively guide the general public to use basic drugs first. The industry said that the timely adjustment of the catalog is conducive to its better coverage of the patient population, more in line with clinical needs. At the same time, the drug coverage of key populations and diseases should be increased (5).

In 2019, China's health costs accounted for 6.64% of GDP, and drug costs accounted for an average of 40.6% of outpatient-related medical costs and 42.0% of public hospital inpatient costs, The proportion of drug costs has been decreasing year by year (6). The high price of drugs has always been a major concern for patients. And China's drug market management is more difficult, and there have been undesirable behaviors such as excessive waste of medical resources and high drug prices, leading to a series of social problems such as difficulties for patients to use drugs. Since the State Council triggered the “Guidance on Improving Centralized Drug Procurement in Public Hospitals” in 2015, China has carried out five rounds of centralized drug procurement in succession. The centralized procurement of drugs has reduced the price of medical purchases, curbed overcapacity and disorderly competition in medicine, and helped promote social welfare (7). Since December 2018, when the National Health Insurance Bureau organized the pilot drug centralized procurement in 11 cities of “4 + 7”, the results announced from the fifth batch of drug centralized procurement show that a total of 218 drugs have been successfully procured, and a large number of drugs have reduced their prices through centralized procurement, with an average reduction of more than 50%, and the cumulative savings in drug costs have exceeded 150 billion yuan. The cumulative savings in drug costs have exceeded 150 billion yuan (8). The cumulative savings in drug costs have exceeded 150 billion yuan. The new “4 + 7” procurement policy has effectively solved the problem of expensive medical care for residents by cutting off the link between pharmaceutical companies and hospitals, and reducing drug prices to the lowest level while ensuring drug quality. The new “4 + 7” collection policy is a win-win initiative between pharmaceutical companies and patients. However, the new “4 + 7” procurement policy has an impact on the supply of drugs in hospital pharmacy departments. In order to better ensure the supply of drugs in hospital pharmacy departments, we need to strengthen the training of medical personnel, assist drug suppliers to adapt to policy changes, support the research and development of new drugs, and improve the drug procurement trading platform to better ensure the supply of drugs in hospital pharmacy departments (9). The following are some of the initiatives that need to be taken In order to promote the normalization and institutionalization of drug procurement, on January 28, 2021, the General Office of the State Council issued the “Opinions on Promoting the Normalization and Institutionalization of Centralized Drug Procurement”, which requires the inclusion of drugs with high usage and high procurement amounts in the basic medical insurance catalog, and gradually cover all types of clinically necessary and reliable drugs listed in China, so that they can be procured as much as possible. All public medical institutions should participate in the collection, to ensure the accessibility of the masses to drugs, and to promote the formation of a unified open drug collection market, formally normalizing and institutionalizing the collection (10). The national market will be normalized and institutionalized. At the same time, for the implementation of the state in selected equipped with and using drugs in medical institutions, National Health Committee also issued “about national organization appoint drug clinical to use the drug centralized purchasing (11). In order to implement the provision and use of selected drugs in medical institutions, the National Health Care Commission has successively issued the “On the clinical provision and use of selected drugs in the centralized procurement of drugs by national organizations”, “On further improving the clinical provision and use of selected drugs in the centralized procurement of drugs by national organizations (12). The State Health and Welfare Commission has issued the following circulars (13). The notice.

In November 2019, the Medical Security Bureau of Hubei Province issued the Notice on Issuing the Implementation Plan for the Full Implementation of the Pilot Expansion of the Centralized Quantity Procurement and Use of State-Organized Drugs [EHPSF (2019) No. 63], and Wuhan City also issued a work plan for drug collection at the end of 2019 to explore a new model of drug collection in the medical insurance coordination area, and the first batch has been completed. The first batch has already been completed. The first batch in Hubei Province and the provincial-level alliances in 11 provinces and cities (Yu, Yu, E, Xiang, Gui, Qiong, Yun, Qing, Ning, Xinjiang, and Xinjiang Corps) are also being launched (14). However, due to a large number of drug collection platforms, it is not easy to find the right platform. However, there are still many difficulties for medical institutions to complete the task of drug collection in a standardized and efficient way due to many drug collection platforms, short election cycles, frequent catalog changes, and insufficient knowledge of drug collection among medical personnel (15). The outbreak of New Coronary Pneumonia in late 2019 has seriously affected people's lives. Due to the outbreak of Newcastle pneumonia, economies around the world are currently facing severe challenges, with studies proposing that in Europe, the United States, and China, industrial productivity declined and that this decline increased with the severity of the outbreak; and that indices of oil demand, stock markets, GDP growth, and electricity demand decreased significantly with the increase in the severity index of the outbreak (16). However, it has also been suggested that the healthcare sector has shown strong resilience to the epidemic, compared to the transportation, mining, power and heating, and environmental sectors (17). Wuhan, as a city with a high concentration of new crown pneumonia outbreaks, experienced a significantly higher overall price decline than other cities in China (18). At present, many scholars and researchers have conducted relevant studies in China. The World Health Organization (WHO)/Health Action International (HAI) standard survey method was used to investigate the price of basic drugs for children in tertiary children's hospitals in China (19). The study was conducted in Changzhou City, Jiangsu Province on the new antitumor drugs negotiated for national health insurance access (20). The survey was conducted on essential drugs in primary care institutions in Jiangsu Province from 2016 to 2020 (21). The survey was conducted on 16 types of antibacterial drugs in five cities in Hubei Province, China (22). The accessibility of essential drugs in Liaoning Province was analyzed (23). However, there is no separate study reported on Wuhan, Hubei Province, in this context, the accessibility of drugs in Wuhan in this paper is reported below.

## 2. Materials and methods

### 2.1. Investigation time

August 17 – October 17, 2022

### 2.2. Selection of survey areas and institutions

According to the WHO/HAI recommendation that countries should conduct region-wide surveys, the sample areas identified were at least 6, including 1 central administrative area and 5 other administrative areas that are 1 day's drive from the central administrative area. In Wuhan city as the research area, the choice of Wuhan urban area six administrative region, namely Jianghan District, Jiangan District, Hanyang District, Wuchang District, Hongshan District, and Qingshan District. A total of 30 public hospitals and 30 retail pharmacies in the 6 urban areas of Wuhan were studied.

### 2.3. Selection of the type of disease to be investigated

According to the Analysis Report of the Fifth National Health Services Survey in 2013 (24), the top five highly prevalent diseases in China are hypertension, colds, diabetes, gastroenteritis, and cerebrovascular disease in that order) In addition, the incidence of depression has increased rapidly in recent years. Therefore, the scope of this survey is the above six diseases.

### 2.4. Selection of investigational drugs

In order to make the selected drugs comparable among different countries and representative and operational within a country, the survey should include 14 drugs from the global core catalog. On this basis, taking into account the National Essential Drug List (2018 edition) (25), a total of 50 essential drugs were selected for this study.

### 2.5. Evaluation indicators

#### 2.5.1. Accessibility evaluation indicators

Accessibility was calculated as follows: accessibility = the number of institutions where drugs were available/total number of institutions studied × 100%. Evaluation criteria: very low, < 30%; low, 30 to 49%; relatively high, 50 to 80%; high, >80% (26).

#### 2.5.2. Affordability evaluation indicators

The total cost of drugs spent in a certain course of treatment for disease using standard doses of drugs is equivalent to a multiple of the minimum daily wage rate for non-technical staff in government departments (or equivalent to the minimum daily wage rate set by the local human resources and social security department), which according to the 2022 Ministry of Human Resources and Social Security of the People's Republic of China, published data shows that the minimum daily wage in Wuhan is 92.41 RMB (27).

#### 2.5.3. Median price ratio, 25% quartile, and 75% quartile

The Median Price Ratio (MPR) is the ratio of the median price per unit (i.e., per tablet, capsule, milliliter, snap, etc.) of a drug to the International Reference Price (IRP) of that drug. The International Reference Price is calculated and published by Management Sciences for Health (MSH, 2015), a US organization (28). The 25% and 75% quartiles of the median price ratio are used to evaluate the dispersion of the median price ratio. Retail prices in public hospitals should generally not exceed 1.5 times the international reference price (MPR < 1.5); retail pharmacies should generally not exceed 2 times the international reference price (MPR < 2) (29).

### 2.6. Survey implementation and data statistics

Standardized electronic data collection forms were sent to the relevant public hospitals and retail pharmacies in Wuhan city surveyed to collect information on the availability and prices of the surveyed drugs. To ensure the accuracy and reliability of the data, the data collectors were trained prior to the study on the background of the project study, the study methodology (selection of study area, institution, and drug, etc.), and the methods of data collection and processing. Then, two trained researchers first double-entered the data into the WHO -HAI workbook PartIMSH2011 worksheet using the WHO/HAI standardized method (2008 version), cleaned the data by “Data checker”, verified The data were cleaned by “Data checker”, checked for extreme values or logical errors, analyzed, and the results were submitted to WHO/HAI officials for review and confirmation. Secondly, the data results were filled into the table, and the data were entered and statistically analyzed twice using Excel software.

## 3. Results

### 3.1. Availability of essential drugs in the context of

In public healthcare facilities, the average availability of originator and generic drugs was 26.4 and 42.47%, respectively. In retail pharmacies, the average availability of original and generic drugs was 26.8 and 54.4%, respectively. Analysis of the individual availability of 14 core drugs showed that the average availability of original drugs in retail pharmacies was 25.5% and that of generics was 53.6%, while the average availability of original drugs in public hospitals was 19.05% and that of generics was 40.48%.

We refer to Table 1, which shows the availability of drugs in public hospitals and retail pharmacies. As shown in the table, the availability of selected drugs in both public hospitals and retail pharmacies was low. Among the originator drugs, in public hospitals, the availability of atorvastatin was the highest; while in retail pharmacies, the highest availability was for albendazole.

**Table 1:** Availability of individual medicines in public pharmacies and private pharmacies.

**Name of medicine**	**Public pharmacies**		**Private pharmacy**	
	**Availability of** **originator drugs (%)**	**Generics availability (%)**	**Availability of** **originator drugs (%)**	**Generics availability (%)**
Acetylsalicylic Acid	90	63	76.7	96.7
Albendazole	66.7	60	96.3	43.3
Amitriptyline	0	50	0	52
Amlodipine	93.3	66.7	88	98.7
Amoxicillin	0	66.7	0	90
Atenolol	0	20	0	23.3
Atorvastatin	96.7	93.3	80	76.7
Azithromycin	60	66.7	73.3	76.7
Bisoprolol	26.7	66.7	73.3	93.3
Captopril	0	46.7	0	90
Cefalexin	0	36.7	0	86.7
Ceftriaxone injection	60	30	0	26.7
Cefuroxime	46.7	40	23.3	66.7
Cetirizine	60	63.3	23.3	76.7
Chlorphenamine Maleate	0	46.7	0	56.7
Ciprofloxacin	0	33.3	0	40
Clarithromycin	23.3	36.7	16.7	43.3
Clomipramine	0	30	0	33.3
Clopidogrel Bisulfate	56.7	46.7	66.7	63.3
Co-trimoxazole suspension	0	0	0	0
Diazepam	0	0	0	3.3
Diclofenac	60	50	66.7	46.7
Digoxin	46.7	16.7	0	40
Diphenhydramine	0	30	0	23.3
Enalapril	26.7	33.3	10	46.7
Erythromycin	0	23.3	0	30
Fluconazole	16.7	20	10	26.7
Fluoxetine	30	36.7	13.3	26.7
Gliclazide	0	43.3	6.7	43.3
Glimepiride	30	46.7	50	36.7
Irbesartan	46.7	56.7	50	46.7
Levofloxacin	0	43.3	0	56.7
lisinopri	0	20	0	30
Loratadine	36.7	56.7	70	73.3
Losartan	50	53.3	70	76.7
Mebendazole	13	0	26.7	16.7
Metformin	43.3	50	43.3	63.3
Metronidazole	0	56.7	0	60
Mupirocin	36.7	50	36.7	70
Nifedipine	0	30	10	80
Nimodipine	33.3	46.7	56.7	46.7
Omeprazole	46.7	40	66.7	73.3
Oseltamivir	40	46.7	20	60
Paracetamol suspension	0	0	0	0
Promethazine	0	63.3	0	70
Propranolol HCl	0	33.3	0	53.3
Salbutamol inhaler	0	46.7	20	63.3
Sertraline	53.3	53.3	53.3	60
Simvastatin	30	56.7	53.3	70
Tinidazole	0	26.7	0	40

As shown in Table 2 lists the public hospital and drug supply situation of retail pharmacies. 28 originator drugs were found in public hospitals, 30 originator drugs were found in retail pharmacies, 47 generic drugs were found in public hospitals and 49 generic drugs were found in retail pharmacies. In public hospitals, only 10 originators and 15 generics were available at more than 50%. In retail pharmacies, only 13 originator drugs and 26 generics had availability above 50%.

**Table 2:** Availability of medicines in the public sector and the private sector.

**Availability**	**Public facilities**		**Private pharmacies**	
	**Originator brand**	**Lowest-priced** **generic**	**Originator brand**	**Lowest-priced** **generic**
Medicines not found in any outlets	22	3	20	1
Medicines found in fewer than 25% of outlets	3	5	10	4
Medicines found in 25 to 50% of outlets	15	27	7	19
Medicines found in 50 to 75% of outlets	7	14	10	15
Medicines found in more than 75% of outlets	3	1	3	11

### 3.2. The price ratio of essential drugs

#### 3.2.1. Prices in public hospitals

The results in Table 3 show that half of the generic drugs in public hospitals sell for more than twice the international reference price. A quarter of them sells for 9.55 times (75th percentile) or more than the reference price, which indicates that the 25th percentile is 0.82, which means that 25% of the drugs have an MPR of 0.82 or lower i.e., < 1, indicating that the procurement system works efficiently and that the majority of the government obtains a procurement price lower than the international reference price. There was a significant difference between the minimum MPR of 0.03 (propranolol) and the maximum MPR of 83.47 (atenolol).

**Table 3:** Prices in the public sector compared to international reference prices.

	**Lowest-priced generic** **(times)**	**OB** **(times)**
MPR	2.23	28.71
25th percentile MPR	0.82	4.32
75th percentile MPR	9.55	19.56
Minimum MPR	0.03	1.54
Maximum MPR	83.47	58.75

#### 3.2.2. Prices in retail pharmacies

**Figure 1:**
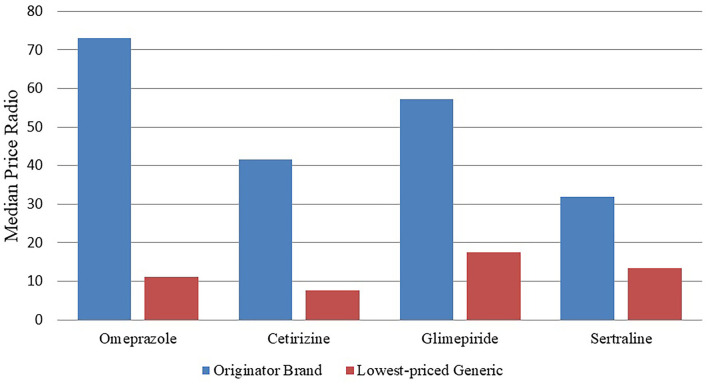
MPRs of these four medicines in the private sector.

We can see Table 4 and Figure 1, where the results in Table 4 were known that the retail pharmacy prices are much higher than the international reference prices. Among the original drugs, omeprazole has the highest price, which is more than 73 times the international reference price. The four original brand products, Glimepiride, Cetirizine, and Sertraline hydrochloride, are more than 35 times more expensive than their international reference prices, and they also have the highest-priced generics.

**Table 4:** Prices in the private sector compared to international reference prices.

	**Lowest-priced generic** **(times)**	**OB (times)**
MPR	3.59	29.24
25th percentile MPR	6.36	4.91
75th percentile MPR	10.16	23.97
Minimum MPR	5.33	1.20
Maximum MPR	14.69	112.75

Table 5, where the results show that the price of original drugs for outpatients in public hospitals is 29.24 times higher than the national reference price in retail pharmacies, while the price of generic drugs in retail pharmacies is 3.59 times higher than the national reference price than in public hospitals.

**Table 5:** MPRs for medicines found in both public and private pharmacies.

**Product type**	**MPR public sector patient prices**	**MPR private sector patient prices**	**% difference between private to public**
Original Research Drug	29.24	28.71	−1.81%
Generic drugs	2.23	3.59	60.99%

### 3.3. Affordability

Affordability was calculated using median prices collected during the survey.Table 6 shows the affordability associated with seven common diseases. Both in public, and retail pharmacies, prices for originator drugs are typically less affordable than the lowest-priced generic drugs. However, in retail pharmacies, some treatments are expensive. For example, treating a peptic ulcer with omeprazole requires a 3.6-day paycheck.

**Table 6:** Affordability of core medicines for common diseases in the public sector and private sector.

**Disease condition**	**Drug name**	**Product type**	**No. of units** **a day**	**Duration** **(days)**	**Day's wage** **(Public pharmacies)**	**Day's wage** **(Private pharmacies)**
Asthma	Salbutamol	Originator	200	as needed		0.24
		Lowest-priced	200	as needed	0.18	0.32
Diabetes	Metformin	Originator	3	30	0.62	0.79
		Lowest-priced	3	30	0.15	0.10
Hypertension	Captopril	Originator	2	30		0.71
		Lowest-priced	2	30	0.05	0.02
	Bisoprolol	Originator	2	30	1.73	1.84
		Lowest-priced	2	30	0.38	1.16
Hypercholesterolaemia	Simvastatin	Originator	1	30	1.45	2.18
		Lowest-priced	1	30	0.17	0.89
Depression	Amitriptyline	Originator	3	30		
		Lowest-priced	3	30	0.16	0.34
Adult respiratory infection	Amoxicillin	Originator	3	7		
		Lowest-priced	3	7	0.05	0.05
	Ciprofloxacin	Originator	2	7		
		Lowest-priced	2	7	0.05	0.05
	Ceftriaxone	Originator	1	1	0.39	
		Lowest-priced	1	1	0.32	0.02
Ulcer	Omeprazole	Originator	1	30	2.41	3.61
		Lowest-priced	1	30	0.16	0.46

## 4. Discussion

This study is the first to apply the WHO/HAI methodology to the accessibility of essential drugs in Wuhan, Hubei Province, China. Not only does it provide a complete report on the availability, price, and affordability of 50 essential drugs in the six urban areas surveyed in Wuhan, China: The availability showed that the availability of original drugs was average in public hospitals and retail pharmacies, and the availability of generic drugs was higher in retail pharmacies, but average in public hospitals. The prices of both original and generic drugs were lower in public hospitals than in retail pharmacies. The affordability results provide us with an analysis of drug affordability for six common diseases, most of which are affordable except for very few, indicating that people's living standards are improving. It also reveals some problems in the accessibility and price of drugs in Wuhan, and we analyze the problems that arise as follows.

First, according to the criteria recommended by the WHO/HAI survey, the average availability results of 50 essential drugs in the six regions of Wuhan city surveyed were: the availability of essential drugs in Wuhan is far below the standard of 80% set by WHO, which is relatively unsatisfactory. This is in line with previous scholarly research: in middle and high-income countries (30) and the drug accessibility survey in Nanjing, China (31). The finding that generic drugs are better but availability is still unsatisfactory is consistent with what emerged in previous scholarly studies: in middle- and high-income countries and in our Nanjing drug accessibility survey. The reasons for the low accessibility include: API shortage is one of the main reasons leading to shortages of essential medicines, some basic drug preparations are in short supply due to shortage of active pharmaceutical ingredients (32). As API producer in China, and its scale is mainly thanks to lower the production cost, and the low cost of production at the expense of the environment pollution. Along with our country continue to strengthen environmental management, active pharmaceutical ingredients industry pressure surge, objectively restricts its development. To a certain extent caused API manufacturers are racing to profit-seeking frequent “monopoly” phenomenon, API prices caused by the natural increase, the essential drugs production cost increase, enterprise capacity, forcing suppliers price is higher than the price, eventually led to the grassroots medical institutions to purchase. Secondly, as the core part of the supply chain, under the condition of normal supply meet API, if there is a willingness to manufacture can guarantee the timely supply determines the basic drugs. Starting from the system itself, the basic drug pricing, reimbursement, with small profit with quantity in the price for the principle, but the benefit maximization is the basic purpose of enterprise management, therefore pharmaceutical enterprise production cost and the unequal of the growing contradiction of the bid price. Another in order to obtain profits, some companies even through the lower the quality of raw materials, reduce drug active ingredient, simplify the production process to reduce the cost, led to the drug's potential quality hidden trouble (33). At the same time, the low profit forcing some production enterprise production or switch to other drugs, directly affect the supply of basic drugs.

Second, in Wuhan City, the drugs investigated showed easier access to generic drugs in both public hospitals and retail pharmacies. This is similar to the previous situation of overall essential drugs in China (34). We analyze the following reasons. First, in terms of the actual market situation, since the technical requirements and cost investment of originator drugs are significantly higher than those of generics at both the R&D and commercial stages, and also bear higher risks than generics, generic drugs are still dominant in the results formed by pharmaceutical R&D (35). Second, the “4 + 7” collection policy (the “4 + 7” collection policy is a new type of drug management policy to ensure the development of health care for residents, including the initiators of China's Health Insurance Bureau, the National Health Commission, the State Drug Administration, of which “4 + 7” includes 4 municipalities directly under the central government (Beijing, Tianjin, Shanghai, Chongqing), 7 sub-provincial cities (Shenzhen, Guangzhou, Xiamen, Chengdu, Xi'an, Shenyang, Dalian), the above 11 cities as the implementation of the pilot (36). After the implementation of the policy, generic drugs have effectively replaced the original drugs. After the implementation of the policy, some researchers found that the proportion of generic drugs in the ‘4 + 7' list rose from 60.73 to 77.80%; that is, 17.08% of the original drugs were replaced by generic drugs in the pilot cities (37). And at the end of 2019, Wuhan was the first city in China to explore the centralized procurement of non-over-evaluated drugs in the medical insurance co-ordination area and became the first city in China to launch municipal-level drug procurement with quantity. So Wuhan shows the phenomenon that generic drugs are more easily available.

Third, in Wuhan city, the surveyed drugs had higher average availability in retail pharmacies compared with public hospitals, and the price differences between original and generic drugs surveyed in public hospitals and retail pharmacies were significant. We analyze the following reasons: firstly, because of the flexible procurement channels of retail pharmacies, the drug purchasing groups are more complex, and pharmacy operators can choose suitable distribution companies according to their needs, and the variety of drug distribution can more fully and flexibly meet the needs of the drug purchasing groups of retail pharmacies. Secondly, with the implementation of the “zero-rate” sales policy of public hospitals, social pharmacies have more rigid expenses, including personnel, space, and taxes. With the in-depth promotion of the “4 + 7” collection policy, the completion of the tasks of the selected varieties in public hospitals will be included in the performance assessment system (38). The proportion of medical insurance payments for non-selected varieties will also be gradually reduced. The in-hospital share of non-selected original drugs is expected to remain under pressure, and non-selected original drug manufacturers are expected to shift the focus of promotion to out-of-hospital markets, such as social pharmacies. In addition, public hospitals are mandatorily included in participation in the quantity procurement, and the price is in accordance with the unified winning price, while social pharmacies are not mandatorily required, the price is still the implementation of market-regulated prices (39). Finally, patients' awareness of self-medication is increasing, and patients can choose drugs of different manufacturers, sizes or dosage forms according to their own wishes, and retail pharmacy operators can meet the needs of different patients by improving the accessibility of drugs.

Relevant measures should be taken to improve the above situation.

One, the supply of originator drugs is appropriately ensured. This is a synergistic development of many policies, such as health insurance policies and economic development policies, which will lead to equitable access to drugs (40). For example, for the unsuccessful originator drugs, it can be considered to gradually withdraw from the standing supply system of public hospitals, while for the high-end patients who really have personalized needs, they can be diverted to social pharmacies, which is also in line with the health insurance policy orientation of “basic medical care for basic” (41). This is also in line with the health insurance policy direction of “basic medical care and basic protection”. The basic drugs are generic varieties, and only the dosage form and specification are specified, not the manufacturer. The higher price of original drugs has largely affected their use, which is the time for generic drugs to make up for the shortage of supply, but at present, the work of consistency evaluation of generic drugs has not been completed, and a large number of generic drugs cannot be completely replaced with original drugs (42). The efficacy of original drugs is higher than that of generic drugs (43). The original drug is more effective than the generic. If the price of originator drugs can be further controlled within the affordable range through price negotiation and centralized procurement, and the local quality generic drugs can form a benign competition, it can undoubtedly better meet the medical needs and improve the effectiveness of clinical treatment (44). The price of generic drugs can be further negotiated and centralized procurement to control the affordable price of original drugs.

Second, there is a lack of regulation of retail pharmacy prices, and it is important to try to ensure price transparency. According to the National Bureau of Medical Security's “Opinions on the good management of current drug prices (45). The spirit of the document, adheres to the general direction of regulating drug prices by the market. Business enterprises can set their own prices and regulate drug prices according to market conditions. It can be seen: the current pricing policy of drugs sold in private medical institutions is liberalization, which appears to raise the price of drugs, marketing its most profitable drugs. Specifically, a series of institutional measures can be introduced to deepen the reform of pharmaceutical prices and promote the rational formation of prices, it is clear to increase the monitoring of pharmaceutical prices and information dissemination. For example, on March 25, 2020, the Xuancheng City Medical Insurance Bureau of Anhui Province released information on conventional drug prices to the public through Xuancheng Daily and Xuancheng City Medical Insurance Bureau, Xuanzhou District Medical Insurance Bureau, and major information public numbers. As early as February 26, the Xuancheng City Medical Insurance Bureau of Anhui Province published “Monitoring and Information Release of Regular Drug Prices in Designated Pharmacies”. Regardless of the policies and interventions adopted by the state, price transparency is crucial because it empowers the government to procure drugs, gives healthcare providers the power to prescribe, and most importantly, gives patients the power to purchase drugs.

The shortcomings of the study are that only secondary and higher level public health care institutions were selected and primary care institutions were not included, making the sample data biased, and caution is needed regarding extrapolation of the results to a larger geographic area. The survey methodology only required the inclusion of drugs with specific strengths and dosage forms. This may lead to an underestimation of the availability of certain drugs, as some institutions may have stocked other dosage forms or strengths of the investigated drugs on the day of the datacollection. This means that for the same drug, other strengths and dosage forms may be available; however, they were not taken into account when examining the availability and price of the drug. It is expected that the above deficiencies will be addressed in future studies and analyzed in depth to provide more targeted recommendations.

## 5. Conclusion

This study is the first study to assess the availability and price of medicines in Wuhan, Hubei Province: it is valuable and innovative as a policy study to analyze the impact of drug policies on drug procurement and prices; in summary, this study reveals the main barriers to improving equitable access to medicines in Wuhan. Measures are needed to improve equitable access to medicines, including effective and efficient procurement policies, promoting the development of retail pharmacies, and increasing the transparency of drug prices in the retail sector.

## Data availability statement

The original contributions presented in the study are included in the article/supplementary material, further inquiries can be directed to the corresponding author.

## Author contributions

The conception and design of this study were primarily conducted by ZD and SZ. Data collection is made up of GS. The drafting of the article was mainly the responsibility of SZ. All authors have reviewed the analysis, interpretation of the data, contributed to the drafting of the manuscript, revised the manuscript for important intellectual content, approved the final version to be published, and agree to be accountable for all the aspects of this study.
